# Identification of a novel lipid metabolism-related gene signature within the tumour immune microenvironment for breast cancer

**DOI:** 10.1186/s12944-022-01651-9

**Published:** 2022-05-13

**Authors:** Xu Chang, Peng Xing

**Affiliations:** Department of Surgical Oncology, Breast Surgery, General Surgery, First Affiliated Hospital of China Medical University, No.77 PuHe Road, Shenyang North New Area, Shenyang, 110122 China

**Keywords:** Breast cancer, Lipid metabolism, Tumour immune microenvironment, Immune-related analysis, Immunotherapy

## Abstract

**Background:**

Systemic factors can strongly affect how tumour cells behave, grow, and communicate with other cells in breast cancer. Lipid metabolic reprogramming is a systemic process that tumour cells undergo; however, the formation and dynamics of lipids associated with the tumour immune microenvironment (TIME) remain unclear. The investigation of the sophisticated bidirectional crosstalk of tumour cells with cancer metabolism, gene expression, and TIME could have the potential to identify novel biomarkers for diagnosis, prognosis, and immunotherapy. This study aimed to construct a prognostic signature to detect the bicrosstalk between the lipid metabolic system and the TIME of breast cancer.

**Methods:**

To detect the expression of LRGs and execute GO/KEGG analysis, the R program was chosen. Considering the clinical information and pathological features, a prognostic gene signature was constructed by LASSO Cox regression analysis. TMB, MSI, and immune infiltration analyses were performed, and consensus cluster analysis of LRGs was also performed.

**Results:**

These 16 lipid metabolism-related genes (LRGs) were mainly involved in the process of lipid metabolism and fatty acid binding in breast cancer. Prognosis analysis identified the prognostic value of FABP7(Fatty acid binding protein 7) and NDUFAB1(NADH:ubiquinone oxidoreductase subunit AB1) in breast cancer patients. The prognostic gene signature constructed with FABP7 and NDUFAB1 was significantly related to immune cell infiltration and could predict the overall survival rate with above average correctness of breast cancer patients. FABP7 and NDUFAB1 were proven to have relevance in immune cell infiltration and tumour mutation burden (TMB). Consensus cluster analysis identified that the upregulated mRNAs were mostly related to the oncogenesis process, while the downregulated mRNAs were associated with immune-related signalling pathways.

**Conclusion:**

A comprehensive analysis was performed to evaluate the lipid metabolic system and identified a signature constructed by two prognostic genes for immunotherapies in breast cancer. The results also revealed evidence of vulnerabilities in the interplay between the lipid metabolic system and the TIME in breast cancer. Further data with clinical studies and experiments are warranted.

**Supplementary Information:**

The online version contains supplementary material available at 10.1186/s12944-022-01651-9.

## Introduction

Breast cancer has become the most prevalent malignancy worldwide according to the latest statistical results [1]. This highly heterogeneous malignancy, which comprises different subtypes, is still a serious threat to the health of women, whereas the triple-negative breast cancer (TNBC) subtype has always been known to have the worst prognosis [2–4]. Multiple research studies have proven that how tumour cells grow, behave, and communicate with other cells are determined not only by the characteristics of cancer cells but also by their sophisticated surrounding environment [5, 6]. The tumour microenvironment (TME) has been proven to be a dynamic community containing tumour cells and tumour-related cells [7]. The TIME (the tumour immune microenvironment), which represents the immune part of the TME, plays crucial roles, and studies have illustrated the complicated bidirectional crosstalk between tumour cells and the TIME in breast cancer [8].

Reprogramming of energy metabolism that can actively contribute to cancer development has been recognized as one of the cancer hallmarks [9, 10]. Carcinogenic events can alter the regulation of metabolic pathways, which in turn enable the proliferation and survival of cancer cells in the microenvironment by providing selective advantages [11, 12]. Lipid metabolism, including fatty acid metabolism and fatty acid transport, which can be influenced by factors such as age, obesity, menopause, drugs, and diet, is also highly activated in breast cancer cells [13, 14] and can both promote and inhibit the oncogenesis and progression of cancer cells by reassigning nutrients in the microenvironment of breast cancer [15, 16]. FABP7 (Fatty acid binding protein 7) is a member of the FABP intracellular lipid chaperone family that regulates lipid metabolism by increasing fatty acid uptake, FAO, and lipolysis [17]. NDUFAB1 (NADH:ubiquinone oxidoreductase subunit AB1), on the other hand, is a mitochondrial acyl carrier protein that participates in lipid metabolism by interacting with other mitochondrial proteins [18].

Immunotherapies such as immune checkpoint blockades and other immunotherapeutic strategies have furnished new hopes for breast cancer patients [19, 20]; however, the low response rate limits the application of tumour immunotherapy [21]. Hence, improved analysis of how the lipid metabolic system with the TIME modulates cancer development and evasion from tumour-suppressive surveillance may reveal clues for novel anticancer immunotherapeutic strategies directed at lipid metabolic targets.

Therefore, in this study, 16 lipid metabolism-related genes (LRGs) were selected to detect the bidirectional interplay of the lipid metabolic system of tumour cells with the TIME and construct a prognostic signature to explore the dynamic lipid metabolic signature difference in breast cancer. The results could provide new evidence for identifying novel prognostic biomarkers of immunotherapies for breast cancer and contribute to revealing the heterogeneity of the TIME in breast cancer.

## Materials and methods

### Date and sample source

The RNA-sequencing (RNA-seq) data and clinical information of breast cancer patients were retrieved from The Cancer Genome Atlas (TCGA) database, which was released on June 1, 2021. R software (version 4.0.3) was used to develop all data analysis methods and the R package. All the obtained expression data of breast cancer patients are shown in Table S1 and were normalized to Fregments Per Kilobase per Million (FPKM) values for subsequent investigation. The patients with breast cancer were diagnosed mainly in 2008–2010, and the clinical information were uploaded to TCGA in 2016. The workflow of this study is shown in Fig. S1.

### Identifying the different expressions of LRGs

Sixteen LRGs in total that participate in the lipid metabolic system in breast cancer were selected. The limma and reshape2 R packages were used to detect the difference in LRG expression in breast cancer and normal tissues [22, 23]. The STRING database was used to search the hub genes by the set with a minimum interaction score of 0.9 [24].

### Functional enrichment analysis

To further identify the function of these LRGs in breast cancer, GO and KEGG databases were selected, and the data were analysed by functional enrichment analysis. The GO (Gene Ontology) database is a web-based tool for determining gene function for MF (molecular function), BP (biological pathways), and CC (cellular components) [25]. Gene set enrichment analysis detected gene pathway enrichments in KEGG (Kyoto Encyclopedia of Genes and Genomes), an open resource [26]. To better understand the carcinogenesis of these LRGs, the ClusterProfiler R package (version 3.14.0) was used to analyse the GO functions and KEGG pathways of these potential targets [27].

### Construction of the lipid metabolism-related gene prognostic model

Cox regression analysis was utilised to determine the prognostic significance of the LRGs. The Kaplan–Meier approach was used to calculate the prognostic value of these 16 LRGs, which were then examined using the log-rank test and univariate Cox proportional hazard regression, yielding *P* values and hazard ratios (HRs) with 95% confidence intervals (CIs). LRGs with substantial prognostic value, as demonstrated by Kaplan–Meier survival curves, were chosen. The chi-square test was selected to clarify the correlation between the prognostic LRGs and clinical TNM stage, and the Wilcoxon test was used to explore the correlation of the age factor with the prognostic LRGs. Based on the prognostic value of these LRGs, a prognostic model containing the two prognostic LRGs was developed by LASSO Cox regression analysis for breast cancer patients using 10-fold cross-validation to determine the optimal value of penalty parameter λ. According to the risk score, the patients with breast cancer were separated into two subgroups with low risk and high risk, and the overall survival (OS) possibility between these two groups was compared by the Kaplan–Meier method. Receiver operating characteristic (ROC) analysis was selected to predict the diagnostic accuracy of each gene. Considering the pathological characteristics, a predicted nomogram was developed to predict the 1-year, 3-year, and 5-year overall survival possibility through the forestplot package in R software [28].

### Immune cell infiltration analysis of the prognostic LRGs

The correlation between the prognostic LRGs and immune cell infiltration was evaluated using the ssGESA R package for comprehensive analysis of tumour-infiltrating immune cells in breast cancer. In the analysis of the correlation between the immune checkpoints and the prognostic LRGs, the ggplot2 R package was selected. Spearman’s correlation analysis was chosen for the examination of tumour mutation burden (TMB) and microsatellite instability (MSI), with a *P* value less than 0.05.

### The cluster analysis of LRGs

The raw counts of RNA-sequencing data of patients with breast cancer and accompanying clinical information were received from the TCGA-BRCA cohort, with the collection and application methods complying with the recommendations and rules. The ConsensusClusterPlus R package (v1.54.0) was used for consistency analysis [29], and the pheatmap R package (v1.0.12) was used for clustering heatmaps. Genes with SD > 0.1 were kept in the gene expression heatmap. If the number of input genes was greater than 1000, the SD was sorted, and the top 25% of the genes were extracted. The Limma R package (version: 3.40.2) was used to study the differential expression of mRNAs. The adjusted *P* value was analysed to correct for false-positive results in TCGA or GTEx. The results with “Adjusted *P* < 0.05 and Log (Fold Change) >1 or Log (Fold Change)< −1” were defined as the thresholds for the screening of differential expression of mRNAs. Fold-change numbers and corrected *P value*s were used to create volcano graphs. Hierarchical clustering was used to look for mRNAs that were differentially expressed in tumour and normal tissues.

## Results

### The expression of LRGs in breast cancer

**Fig. 1:**
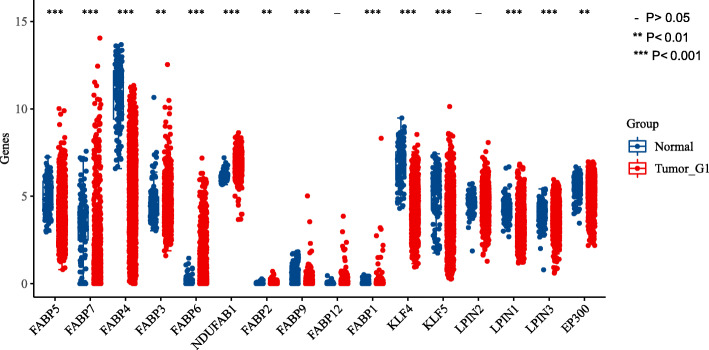
The expression of 16 LRGs in breast cancer and breast tissues, tumour, red; Normal, blue. LRGs, lipid metabolic related genes. Asterisks represent levels of significance, - *P* > 0.05, *P < 0.05, **P < 0.01, ***P < 0.001

The expression levels of 16 LRGs in breast cancer and normal breast tissues were first detected by data from TCGA-breast cancer. A total of two genes showed no significant change in breast cancer (Fig. 1). More specifically, the expression of FABP5, FABP7, FABP3, FABP6, NDUFAB1, FABP2, FABP1, KLF5, LPN1, LPN3, and EP300 was upregulated compared with that in normal tissues, while the expression of FABP4, FABP9, and KLF4 was downregulated (all *P* < 0.05).

### Functional enrichment analysis of LRGs

**Fig. 2:**
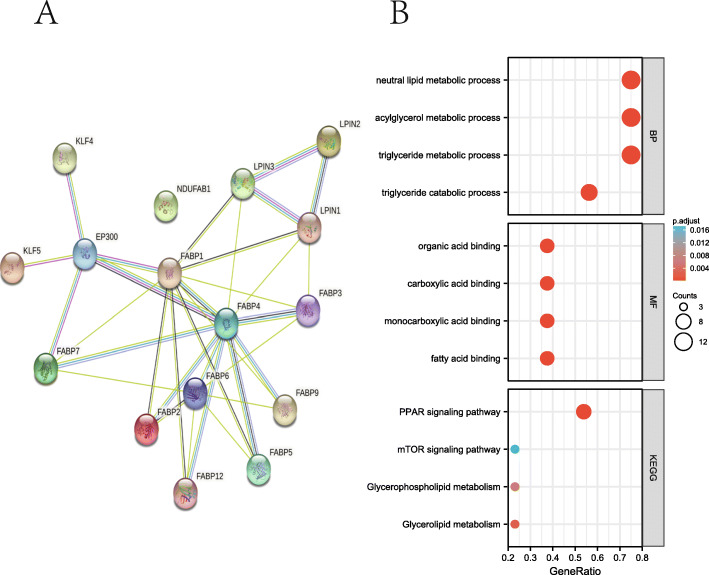
The functional enrichment analysis of LRGs in breast cancer. **A** The PPI network of LRGs using STRING database. **B** The enriched item in gene ontology (GO) analysis and Kyoto Encyclopedia of Genes and Genomes (KEGG) analysis. The size of circles represented the number of genes enriched. PPI, protein-protein interaction; BP, biological process; MF, molecular function

A protein–protein interaction (PPI) network was constructed to explore the correlation of these LRGs, which was set with a minimum interaction score of 0.9, as shown in Table 1. The analysis results revealed that EP300, FABP1, FABP2, FABP4, FABP7, KLF4, and KLF5 were hub genes (Fig. 2A). GO and KEGG databases were selected to screen the function of the LRGs. The results suggested that these 16 LRGs were mainly involved in the positive regulation of the triglyceride metabolic process, acylglycerol metabolic process, neutral lipid metabolic process, triglyceride catabolic process, fatty acid binding, monocarboxylic acid binding, carboxylic acid binding, and organic acid binding in GO analysis. Moreover, the analysis results from the KEGG database revealed that 16 LRGs were primarily concerned with the PPAR signalling pathway, glycerolipid metabolism, glycerophospholipid metabolism, and mTOR signalling pathway (Fig. 2B, Table 2).

**Table 1:** The protein–protein interaction (PPI) network constructed by STRING database to explore the interactions of these LRGs

node1	node2	node1_external_id	node2_external_id	combined_score
EP300	KLF4	ENSP00000263253	ENSP00000363804	0.999
EP300	KLF5	ENSP00000263253	ENSP00000366915	0.991
LPIN3	LPIN1	ENSP00000362354	ENSP00000397908	0.937
FABP2	FABP1	ENSP00000274024	ENSP00000295834	0.926
FABP4	EP300	ENSP00000256104	ENSP00000263253	0.918
EP300	FABP7	ENSP00000263253	ENSP00000357429	0.907
EP300	FABP1	ENSP00000263253	ENSP00000295834	0.906
FABP4	FABP1	ENSP00000256104	ENSP00000295834	0.897
FABP2	FABP6	ENSP00000274024	ENSP00000377549	0.876
LPIN2	LPIN3	ENSP00000261596	ENSP00000362354	0.858
FABP1	FABP3	ENSP00000295834	ENSP00000362817	0.841
FABP4	FABP6	ENSP00000256104	ENSP00000377549	0.831
LPIN2	LPIN1	ENSP00000261596	ENSP00000397908	0.815
FABP3	FABP6	ENSP00000362817	ENSP00000377549	0.808
FABP1	FABP5	ENSP00000295834	ENSP00000297258	0.719
FABP4	LPIN3	ENSP00000256104	ENSP00000362354	0.695
FABP4	FABP2	ENSP00000256104	ENSP00000274024	0.695
FABP4	FABP5	ENSP00000256104	ENSP00000297258	0.673
FABP4	FABP3	ENSP00000256104	ENSP00000362817	0.667
FABP4	FABP7	ENSP00000256104	ENSP00000357429	0.659
FABP4	FABP12	ENSP00000256104	ENSP00000353650	0.657
FABP4	FABP9	ENSP00000256104	ENSP00000368362	0.655
FABP12	FABP6	ENSP00000353650	ENSP00000377549	0.654
FABP9	FABP6	ENSP00000368362	ENSP00000377549	0.628
FABP1	FABP7	ENSP00000295834	ENSP00000357429	0.607
FABP7	FABP6	ENSP00000357429	ENSP00000377549	0.588
FABP5	FABP6	ENSP00000297258	ENSP00000377549	0.584
FABP1	FABP9	ENSP00000295834	ENSP00000368362	0.532
FABP3	LPIN1	ENSP00000362817	ENSP00000397908	0.517
FABP4	LPIN1	ENSP00000256104	ENSP00000397908	0.505
FABP1	LPIN3	ENSP00000295834	ENSP00000362354	0.502
FABP1	LPIN1	ENSP00000295834	ENSP00000397908	0.448
FABP1	FABP12	ENSP00000295834	ENSP00000353650	0.43

**Table 2:** Pathway enrichment analysis items of 16 LRGs from KEGG

ONTOLOGY	ID	Description	GeneRatio	BgRatio	*P* value	P.adjust	qvalue
BP	GO:0006641	triglyceride metabolic process	10/11	105/18670	2.23e-22	9.62e-20	5.50e-20
BP	GO:0006639	acylglycerol metabolic process	10/11	129/18670	1.90e-21	2.96e-19	1.69e-19
BP	GO:0006638	neutral lipid metabolic process	10/11	130/18670	2.06e-21	2.96e-19	1.69e-19
BP	GO:0019433	triglyceride catabolic process	8/11	35/18670	1.06e-20	1.14e-18	6.52e-19
BP	GO:0046461	neutral lipid catabolic process	8/11	43/18670	6.51e-20	4.68e-18	2.67e-18
MF	GO:0005504	fatty acid binding	5/11	34/17697	8.82e-12	3.53e-10	1.30e-10
MF	GO:0033293	monocarboxylic acid binding	5/11	64/17697	2.40e-10	4.79e-09	1.77e-09
MF	GO:0005324	long-chain fatty acid transporter activity	3/11	11/17697	2.94e-08	3.92e-07	1.44e-07
MF	GO:0031406	carboxylic acid binding	5/11	193/17697	6.42e-08	5.18e-07	1.91e-07
MF	GO:0036041	long-chain fatty acid binding	3/11	14/17697	6.48e-08	5.18e-07	1.91e-07
KEGG	hsa03320	PPAR signaling pathway	6/9	78/8076	5.48e-11	1.70e-09	1.50e-09
KEGG	hsa04975	Fat digestion and absorption	2/9	43/8076	9.74e-04	0.015	0.013
KEGG	hsa00561	Glycerolipid metabolism	2/9	61/8076	0.002	0.020	0.018
KEGG	hsa00564	Glycerophospholipid metabolism	2/9	98/8076	0.005	0.038	0.034
KEGG	hsa04150	mTOR signaling pathway	2/9	155/8076	0.012	0.075	0.066

### Construction of the prognostic gene model

**Fig. 3:**
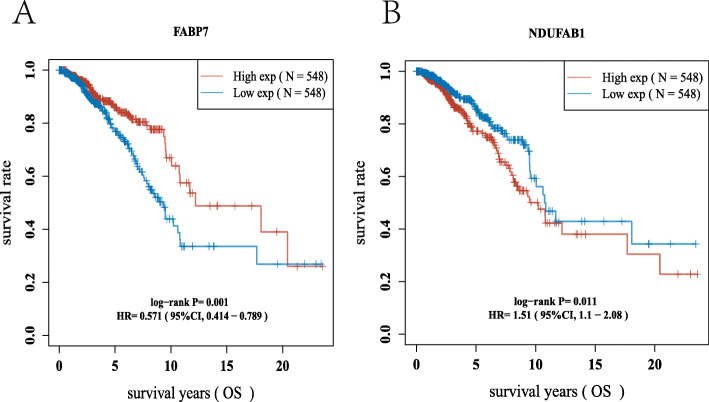
The prognostic value of LRGs in breast cancer. The overall survival curve of **A** FABP7 **B** NDUFAB1 in breast cancer patients in the high-/low-expression group

**Fig. 4:**
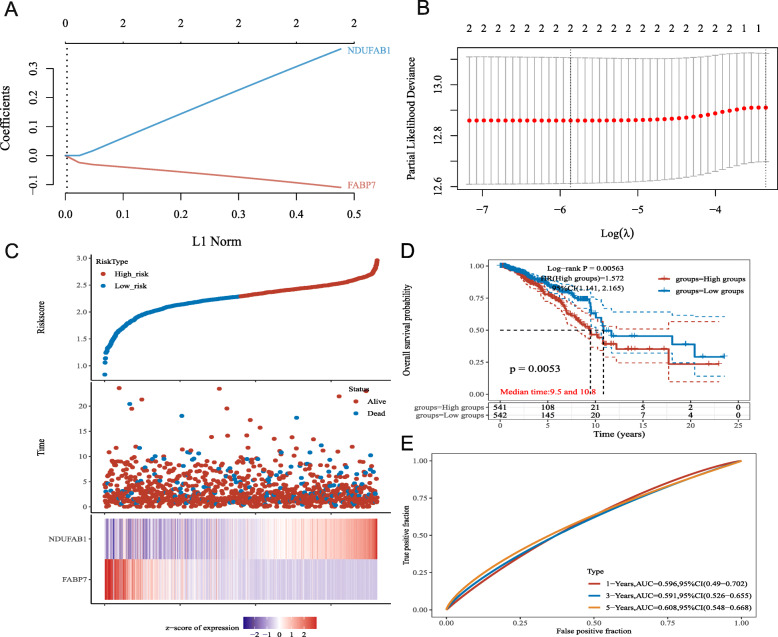
Construction of a prognostic LRGs model. **A** LASSO coefficient profiles of the two LRGs. **B** Plots of the ten-fold cross-validation error rates. **C** Distribution of risk score, survival status, and the expression of 2 prognostic LRGs in breast cancer. **D-E** Overall survival curves for breast cancer patients in the high-/low-risk group and the ROC curve of measuring the predictive value

To clarify the prognostic value of these LRGs, a prognostic gene model was built using univariate Cox regression analysis. The findings revealed that two LRG genes were identified as having prognostic significance, and the Kaplan–Meier survival curves are displayed in Fig. 3. The findings of the prognostic analysis revealed that individuals with breast cancer who had FABP7 downregulation had a low chance of survival (Fig. 3A, *P* = 0.001) and upregulation of NDUFAB1 (Fig. 3B, *P* = 0.011). Based on the prognostic value of FABP7 and NDUFAB1 in breast cancer, a prognostic gene signature containing these two LRGs (FABP7 and NDUFAB1) was constructed by LASSO Cox regression analysis (Fig. 4A, B), and the final results were calculated with the formula risk score = (− 0.1013) * FABP7 + (0.3367) * NDUFAB1. Based on the risk score, the two groups were separated into high-risk and low-risk groups. The distribution of risk scores, survival status, and expression of these two genes were all present. As the risk score increased, the risk of death of breast cancer patients increased, while the survival time decreased (Fig. 4C). The Kaplan–Meier survival curves showed that breast cancer patients with high-risk scores (median time = 9.5 years) had a worse overall survival (OS) rate than those with low-risk scores (median time = 10.8 years) (*P* = 0.0053), and the 1-year, 3-year, and 5-year ROC curves had AUCs of 0.596, 0.591, and 0.608, respectively (Fig. 4D, E).

### The construction of the predictive nomogram

**Fig. 5:**
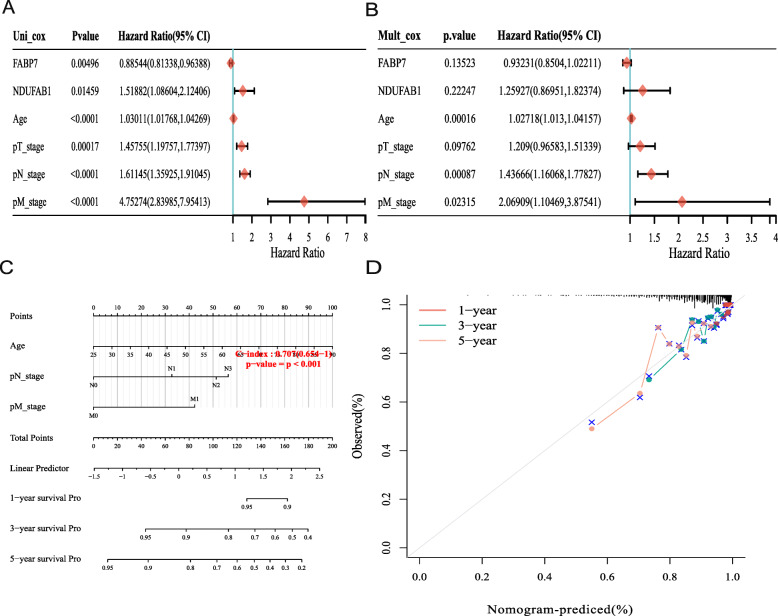
Construction of a predictive nomogram. **A-B** Hazard ratio and *P* value of the constituents involved in univariate and multivariate Cox regression considering clinical the parameters and two prognostic LRGs in breast cancer. **C-D** Nomogram to predict the 1-year, 3-year, and 5-year overall survival rate

Allowing for the correlation between pathological features and these two prognostic LRGs (FABP7 and NDUFAB1), a predictive nomogram was subsequently built to predict the survival probability. By analysing the results, univariate analyses identified the expression of FABP7 and NDUFAB1, and the stage of pT, pN, and pM were the factors that could affect the prognosis of breast cancer patients. More interestingly, the univariate and multivariate analyses illustrated that age was the factor affecting prognosis, and the results are shown in Fig. 5A-B. After analysing the expression of FABP7 and NDUFAB1 with clinical characteristics, the results demonstrated that the expression of FABP7, as well as NDUFAB1, was significantly correlated with T stage and age, and the results are shown in Tables 3 and 4. Furthermore, the 3- and 5- year overall survival (OS) rates in the complete cohort could be predicted reasonably well by assessing the predictive nomogram data, as shown in Fig. 5C, D.

**Table 3:** The correlation between the clinical characteristics and the expression of FABP7

Characteristic	Low expression of FABP7	High expression of FABP7	*P* value	Method
n	541	542		
T stage, n (%)			0.014	Chisq.test
T1	125 (11.6%)	152 (14.1%)		
T2	325 (30.1%)	304 (28.1%)		
T3	64 (5.9%)	75 (6.9%)		
T4	25 (2.3%)	10 (0.9%)		
N stage, n (%)			0.694	Chisq.test
N0	247 (23.2%)	267 (25.1%)		
N1	177 (16.6%)	181 (17%)		
N2	62 (5.8%)	54 (5.1%)		
N3	40 (3.8%)	36 (3.4%)		
M stage, n (%)			0.258	Chisq.test
M0	448 (48.6%)	454 (49.2%)		
M1	13 (1.4%)	7 (0.8%)		
Age, meidan (IQR)	61 (50, 70)	55 (47, 64)	< 0.001	Wilcoxon

**Table 4:** The correlation between the clinical characteristics and the expression of NDUFAB1

Characteristic	Low expression of NDUFAB1	High expression of NDUFAB1	*P* value	Method
n	541	542		
T stage, n (%)			0.014	Chisq.test
T1	159 (14.7%)	118 (10.9%)		
T2	296 (27.4%)	333 (30.8%)		
T3	71 (6.6%)	68 (6.3%)		
T4	13 (1.2%)	22 (2%)		
N stage, n (%)			0.908	Chisq.test
N0	262 (24.6%)	252 (23.7%)		
N1	175 (16.4%)	183 (17.2%)		
N2	58 (5.5%)	58 (5.5%)		
N3	40 (3.8%)	36 (3.4%)		
M stage, n (%)			0.041	Chisq.test
M0	457 (49.6%)	445 (48.3%)		
M1	5 (0.5%)	15 (1.6%)		
Age, meidan (IQR)	56 (47, 65)	59 (50, 69)	0.002	Wilcoxon

### The correlation between prognostic LRGs and immune cell infiltration in breast cancer

**Fig. 6:**
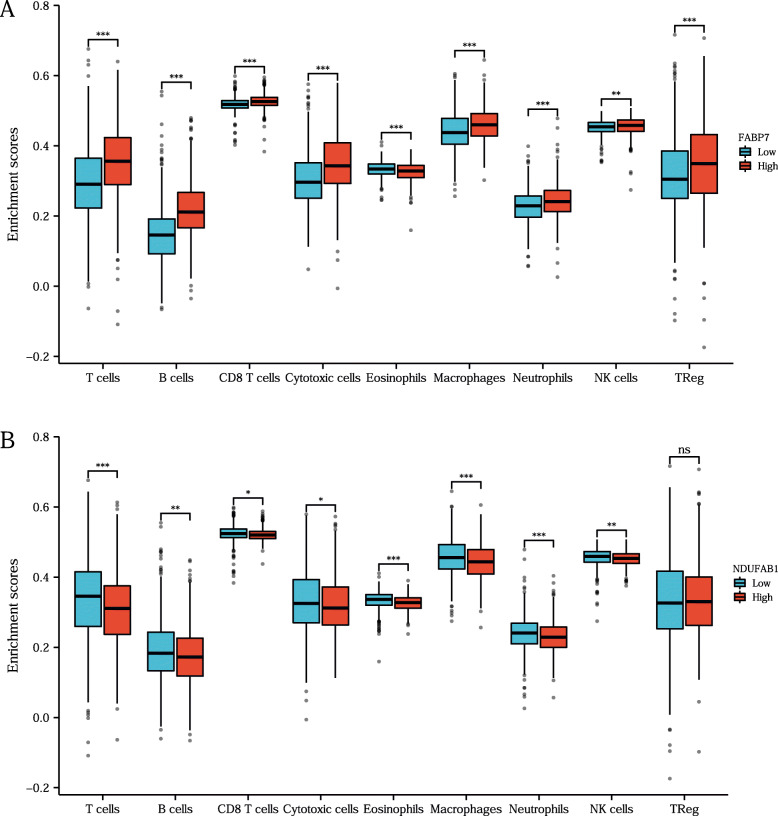
The immune-cell infiltration analysis of the two prognostic LRGs. **A-B** The association between the abundance of immune cells and the expression of FABP7, NDUFAB1 in breast cancer. Asterisks represent levels of significance *P < 0.05, **P < 0.01, ***P < 0.001, ns, no significance

**Fig. 7:**
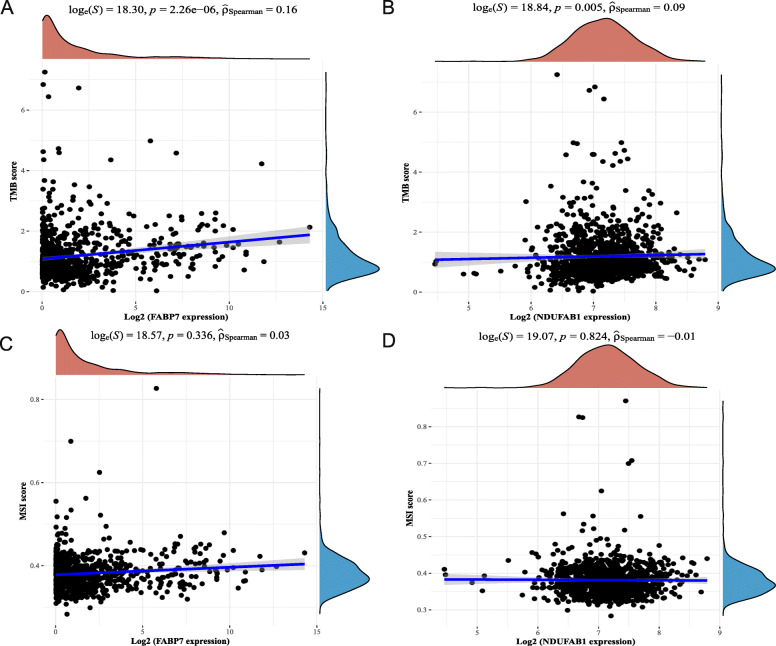
TMB, MSI analysis of the prognostic LRGs (FABP7 and NDUFAB1) in breast cancer. **A-B** The correlation between two prognostic LRGs and TMB in breast cancer. **C-D** The correlation between two prognostic LRGs and MSI in breast cancer. TMB, tumour mutation burden; MSI, microsatellite instability

In this study, correlation analysis for the expression of prognostic LRGs (FABP7 and NDUFAB1) and immune cell infiltration in breast cancer was also performed using the ssGSEA R package. The findings revealed a strong relationship between the expression of prognostic LRGs (FABP7 and NDUFAB1) and the quantity of immunologically infiltrating cells, such as CD8+ T cells, macrophages, neutrophils, cytotoxic cells, eosinophils, NK cells, and Treg cells (Fig. 6 B). A B, all *P <* 0.05). This evidence suggested a significant correlation between the prognostic LRGs and tumour immune infiltration. Moreover, the study detected the correlation between the immune checkpoints (TIGIT, PDCD1, CD274, LAG3, CTLA4) and the prognostic LRGs by the ggplot2 R package, and the results revealed a significant correlation between the immune checkpoints and the two prognostic LRGs (Fig. 7 A B, all *P* *< 0.05*).

### TMB and MSI analysis of LRGs

**Fig. 8:**
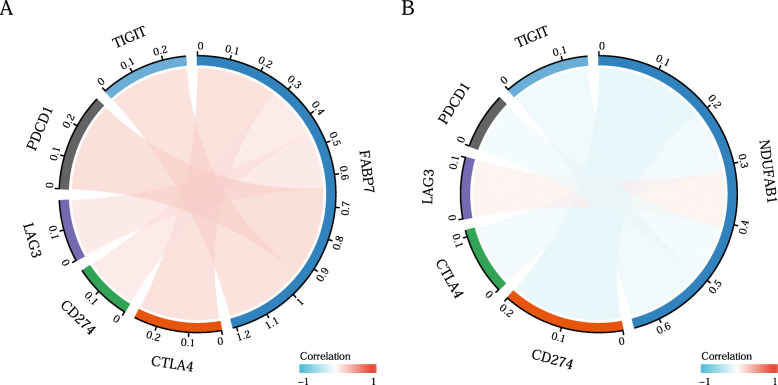
The correlation of the immune-checkpoints(TIGIT, PDCD1, LAG3, CD274, CTLA4) with **A**, FABP7; **B**, NDUFAB1, all *P* < 0.05

Tumour mutation burden (TMB), as well as microsatellite instability (MSI) analysis, could be utilized to anticipate the immunotherapeutic efficacy of breast cancer treatment. To clarify whether these two prognostic LRGs could serve as biomarkers for immunotherapy, the correlation between the two prognostic LRGs and TMB as well as MSI in breast cancer was analysed. The results indicated a favourable relationship between TMB and FABP7 (Fig. 8A, *P* = 2.26e− 06) and NDUFAB1 (Fig. 8B, *P* = 0.005). The findings demonstrated that prognostic LRGs were strongly linked to tumour immune cell infiltration and could serve as biomarkers of immunotherapies for breast cancer.

### Tumour immune infiltration analysis of the prognostic gene model

**Fig. 9:**
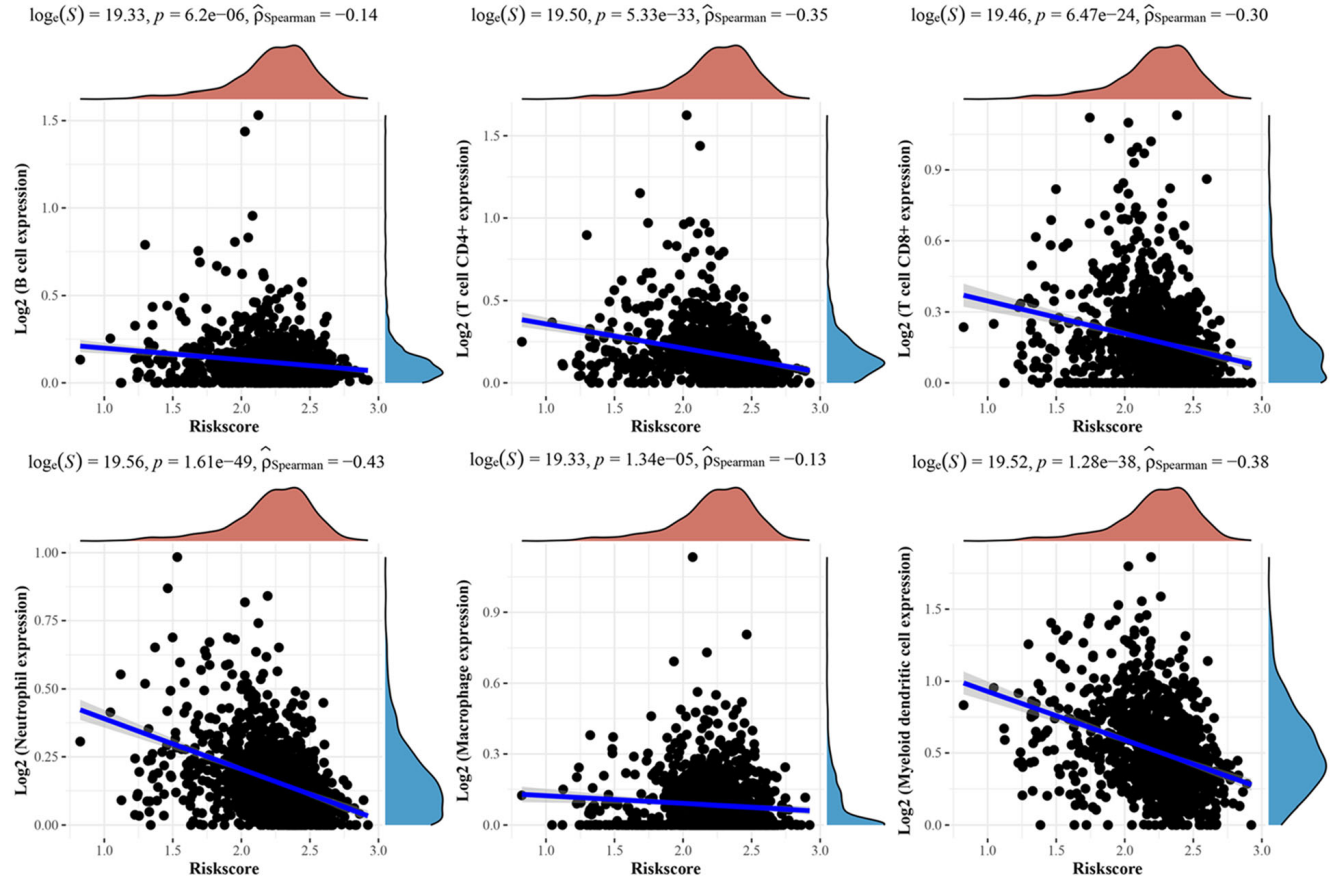
Immune-cell infiltration analysis of the prognostic signature containing two prognostic LRGs (FABP7 and NDUFAB1)

To further screen the correlation of the gene prognostic model containing the prognostic LRGs (FABP7 and NDUFAB1) with the tumour immune microenvironment (TIME) in breast cancer, the ssGSEA method was selected to perform the immune infiltration analysis of this prognostic signature. The analysis results illustrated a negative correlation between the prognostic model containing FABP7 and NDUFAB1 (Fig. 9). The above results revealed a significant correlation of the gene signature with the TIME in breast cancer.

### Consensus clustering analysis of LRGs in breast cancer

**Fig. 10:**
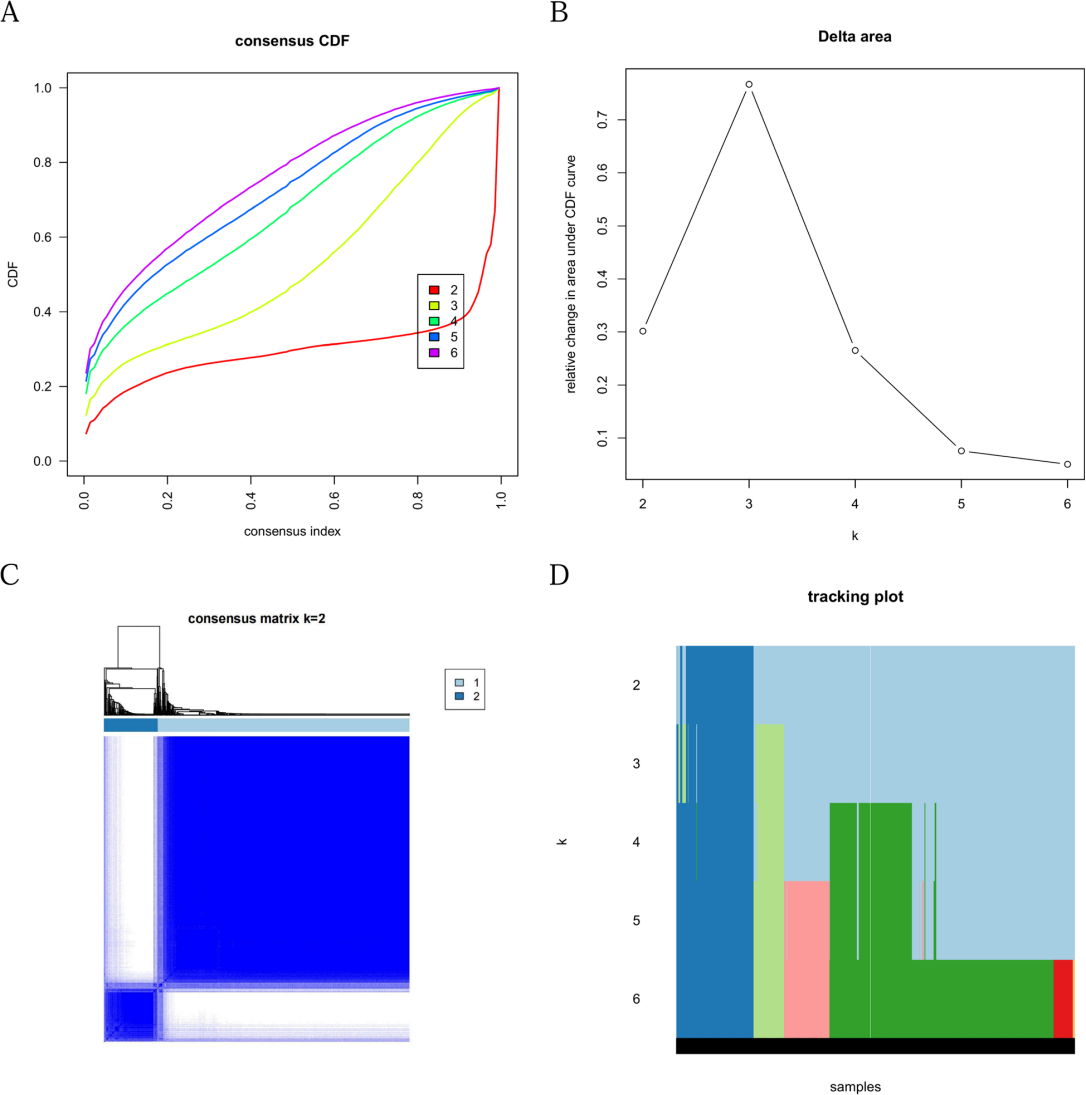
Consensus Clustering Analysis of lipid metabolic-related gene clusters **A-B** Cumulative distribution function (CDF) of consensus clustering by consistency analysis; **C-D** Consensus matrices of the sarcoma patients for k = 2

To explore the different functions of these 16 LRGs, a consensus clustering analysis algorithm was performed. The delta area curve of consensus clustering (Fig. 10A) indicated the relative change in the area under the cumulative distribution function (CDF) curve for each category number k compared with the k–1 consistency analysis (Fig. 10B), and the number of clusters was reduced to two in the end (Fig. 10C, D).

### Functional enrichment analysis of the consensus clusters

**Fig. 11:**
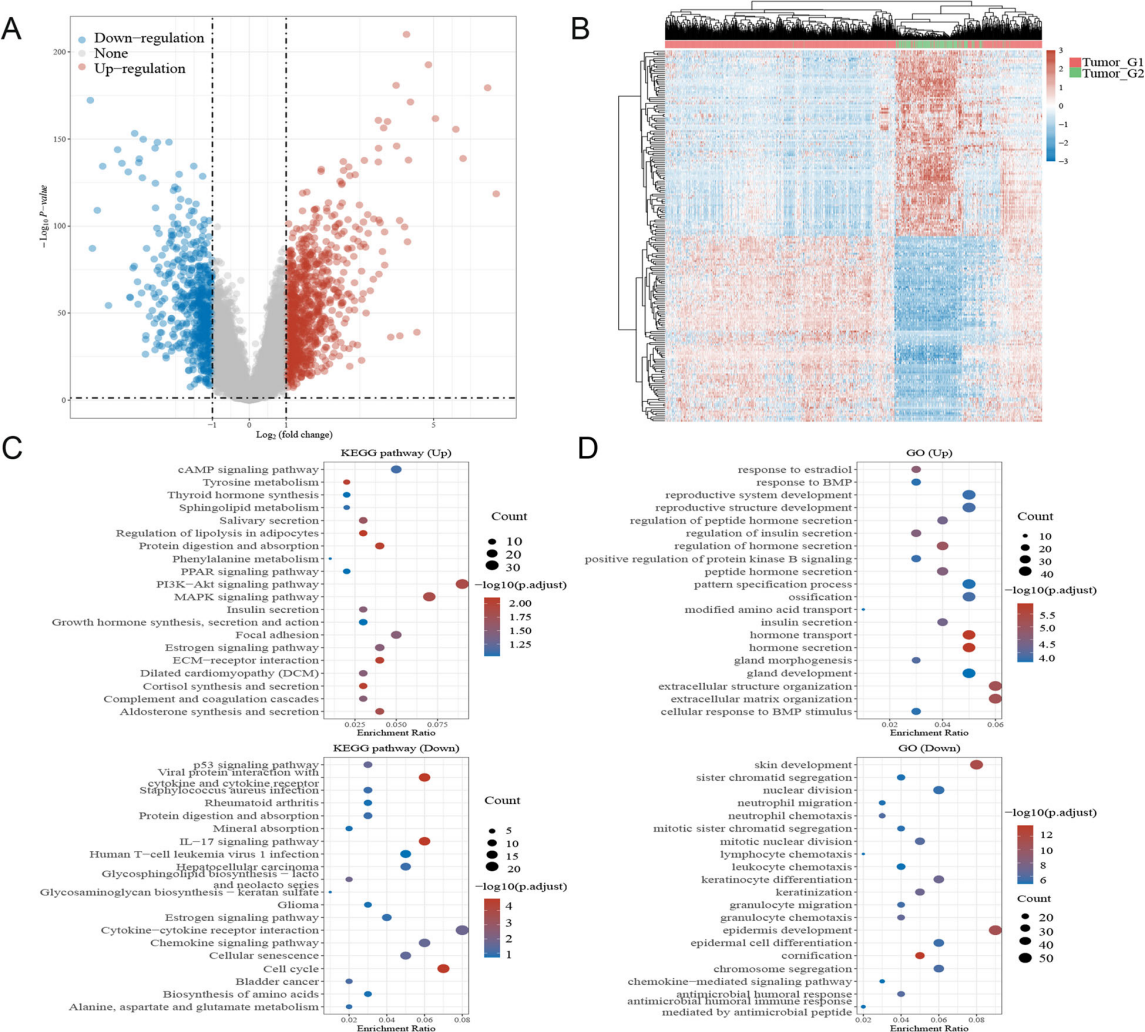
**A** Volcano plots of clustering analysis of mRNAs, **B** Hierarchical clustering analysis of mRNAs, **C-D** The enriched item in gene ontology (GO) analysis and Kyoto Encyclopedia of Genes and Genomes (KEGG) analysis of consensus clusters. The size of circles represented the number of genes enriched

Volcano plots were constructed using fold-change values and adjusted *P* values. The red point in the plot represents the overexpressed mRNAs, and the blue point indicates the downregulated mRNAs with statistical significance. The results revealed that 846 mRNAs were upregulated and 255 mRNAs were downregulated (Fig. 11A). Analysis of mRNAs that were differentially expressed between tumour and normal tissues was conducted using hierarchical clustering (Fig. 11B).

The data were evaluated using functional enrichment analysis via GO/KEGG techniques to further establish the underlying function of putative targets of these two clusters in breast cancer, and the findings with *P* < 0.05 or FDR < 0.05 were judged to be enriched in a meaningful pathway. The analysis results suggested that the up consensus cluster mRNAs were related to the PI3K − Akt signalling pathway, MAPK signalling pathway, insulin secretion, positive regulation of protein kinase B signalling, regulation of hormone secretion, regulation of insulin secretion, and reproductive system development (Fig. 11C). Meanwhile, the down cluster was closely related to the p53 signalling pathway, cell cycle, chemokine signalling pathway, lymphocyte chemotaxis, and IL − 17 signalling pathway (Fig. 11D). More interestingly, both consensus clusters were involved in the oestrogen signalling pathway.

The above results showed the different functions and signature pathways of the consensus clusters of LRGs in breast cancer, which may suggest that the process of the lipid metabolic system comprises lipid formation and dynamics and is related to different biological functions in breast cancer.

## Discussion

Breast cancers, including different subtypes, have always threatened the health of women worldwide, and this is especially true for triple-negative breast cancer (TNBC) [30]. Numerous studies of the TME have redefined tumours from simple gatherings of tumour cells to a complicated community composed of multiple surrounding cells [31]. The tumour immune microenvironment (TIME), which represents the TME’s immunological components, plays decisive roles in breast cancer and prospective immunotherapeutic targets [32]. Studies have shown that breast cancer cells can contain naturally processed and distinct exceptional mutations that can be identified by patients’ immune systems [33]. The major objective of immunotherapy is to promote the switch from a protumour to an antitumour impact to maximize the efficacy of antitumour immunity, and accordingly, immunotherapies such as immune checkpoint blockades and others have offered new clinical strategies for breast cancer patients.

Cancer cells have been known to be able to take advantage of the altered metabolic community to maintain their survival and proliferation [34–36]. The altered lipid metabolic process of cancer cells can further reprogram and impact other cells in the tumour microenvironment (TME), which contributes to the regulation of oncogenesis, aggravation, metastasis, and recurrence of breast cancer [37, 38].

In this study, the expression and prognostic value of these 16 lipid metabolism-related genes (LRGs) in breast cancer were first evaluated, and the results showed that the expression of 11 LRGs was increased compared with that in normal tissues, while the expression of 3 LRGs was decreased. Kaplan–Meier survival analysis results identified the prognostic value of FABP7 and NDUFAB1 in breast cancer patients. GO/KEGG functional enrichment analysis was subsequently performed, and the results illustrated that these 16 LRGs were involved in the triglyceride metabolic process, fatty acid binding, PPAR signalling pathway, and mTOR signalling pathway. FABP7, which regulates lipid metabolism, has been found to be upregulated in triple negative breast cancer (TNBC) and to affect TNBC cell death as a metabolic mediator in nutrient-depleted conditions [39]. It’s also a regulator of lipid metabolism reprogramming in HER2+ breast cancer cells, allowing metastatic cells to adapt and survive in the brain microenvironment [17]. In the meantime, NDUFAB1 has been identified as an important endogenous regulator of mitochondrial bioenergetics and a contributor to lipoic acid production [18]. The above results identified that the selected LRGs were related to the process of lipid metabolic reprogramming, oncogenesis, and inflammation in breast cancer [40–42], of which the expression of FABP7 and NDUFAB1 could affect the prognosis of patients with breast cancer. Furthermore, as lipid metabolism regulators, FABP7 and NDUFAB1 might have the potential to elucidate the mechanism of breast cancer cell invasion and metastasis.

Based on the prognostic value of FABP7 and NDUFAB1, LASSO Cox regression analysis was used to build a prognostic gene model that revealed that breast cancer patients with high risk scores had a lower overall survival rate than those with low risk scores. The correlation of the clinical characteristics with these two prognostic LRGs (FABP7, NDUFAB1) in breast cancer patients was performed using the chi-square test, and the results revealed that both the expression of FABP7 and NDUFAB1 were significantly related to the T stage of breast cancer patients. Moreover, when compared to an ideal model in the full cohort, the predictive nomogram showed that the 3-year and 5-year overall survival rates could be predicted pretty well. This was the first study to develop a lipid metabolism-related prognostic gene signature in breast cancer patients with clinical data, thus providing us with new alternatives for prognostic prediction in breast cancer.

Tumour mutation burden (TMB) [43], as well as microsatellite instability (MSI) [44] analysis, can be used to predict the response to immunotherapies and have proven to be helpful biomarkers in breast cancer for identifying individuals who will benefit from immunotherapies. Using tumour immune cell infiltration-related analysis, the results illustrated that FABP7 and NDUFAB1 were significantly correlated with tumour immune cell infiltration. More interestingly, by immune cell infiltration analysis of the prognostic signature constructed by FABP7 and NADUFAB1, a more significant correlation with tumour immune cells in breast cancer was demonstrated. The above results illustrated that FABP7 and NDUFAB1 could serve as predictive biomarkers in immunotherapies for breast cancer, which also suggested a significant correlation of FABP7 and NDUFAB1 with the tumour immune microenvironment (TIME).

Another important finding of this study revealed the difference in the function of these 16 LRGs by consistency analysis. To further confirm the underlying function of potential targets of these two consensus clusters in breast cancer, the data were analysed by functional enrichment using the GO and KEGG databases. The analysis results suggested that the up cluster was significantly related to the functions of oncogenesis and metabolic reprogramming processes of breast cancer, such as the PI3K − Akt signalling pathway, MAPK signalling pathway, insulin secretion, and regulation of hormone secretion [45–47]. Meanwhile, the down consensus cluster was closely related to immune-related functions and signalling pathways, such as the IL − 17 signalling pathway and chemokine signalling pathway [48, 49]. More interestingly, both consensus clusters were involved in the oestrogen signalling pathway, which is an important signalling pathway for breast cancer [50, 51]. Studies have proven that disturbances in the lipid metabolic system can modulate the menopausal status of women [52]^,^ which could also lead to the unbalanced distribution of nutrients between tumour cells and immune cells in the tumour microenvironment (TME) [53]. The above results showed the different functions and signature pathways of the consensus clusters of LRGs in breast cancer. The results suggested that the lipid metabolic system might induce the reassignment of nutrients in the tumour microenvironment by the oestrogen signalling pathway, which could also modulate the menopausal status of patients with breast cancer.

### Study strengths and limitations

This current study has certain strengths. This study directly detect the correlation between the lipid metabolism-related genes (LRGs) and the tumour immune cell infiltration in breast cancer, and the functional landscape of these LRGs was also illustrated using consensus analysis. These findings would have crucial implications for breast cancer patients’ clinical treatment, endocrine therapy, chemotherapy, and immunotherapy. There are also some limitations to this research. All studies were carried out with the TCGA-BRCA cohort, and more data with in vivo, in vitro research and clinical studies could be used to corroborate the results.

## Conclusions

In conclusion, a thorough investigation of lipid metabolism-related genes was conducted for patients with breast cancer, and a prognostic signature encompassing two biomarkers (FABP7 and NDUFAB1) was discovered for the application of immunotherapy. The results also provided new perspectives for deciphering the bidirectional interplay between the lipid metabolic system and the tumour immune microenvironment (TIME) in breast cancer. More data and investigations are required to corroborate these findings.

## Supplementary Information

**Additional file 1: Table S1.** Characteristics of patients with breast cancer in the TCGA cohort.

**Additional file 2.**

## Data Availability

The datasets used and/or analysed in this study are available from the corresponding author upon reasonable request.

## Abbreviations

LRGs: Lipid metabolic-related genes
FABP7: Fatty acid binding protein 7
NDUFAB1: NADH:ubiquinone oxidoreductase subunit AB1
TME: The tumour microenvironment
TIME: The tumour immune microenvironment
TNBC: Triple negative breast cancer
HER2: Human epidermal growth factor receptor 2
GO: Gene Ontology
MF: Molecular function
BP: Biological pathways
CC: Cellular components
KEGG: Kyoto Encyclopedia of Genes and Genomes
TMB: Tumour mutation burden
MSI: Microsatellite instability
NK cell: Natural killer cell
