# Supplementary material for: Identification of a novel lipid metabolism-related gene signature within the tumour immune microenvironment for breast cancer
Source: Lipids Health Dis. 2022 May 13;21:43. doi: 10.1186/s12944-022-01651-9 (PMC9103058; doi:10.1186/s12944-022-01651-9)
Supplement: Supplementary file 1 — Additional file 1: Table S1. Characteristics of patients with breast cancer in the TCGA cohort. [file 12944_2022_1651_MOESM1_ESM.docx]

Table. S1 Characteristics of patients with breast cancer in the TCGA cohort

| **Characteristics** |  |  | No.(%) |
| --- | --- | --- | --- |
| **Age** |  |  |  |
| ≥ 60 years |  |  | 641(58.4) |
| < 60 years |  |  | 449(40.9) |
| Not available |  |  | 7(0.6) |
| **Menopause status** |  |  |  |
| Post |  |  | 725(66.1) |
| Pre |  |  | 259(23.6) |
| Peri |  |  | 59(5.4) |
| Indeterminate |  |  | 54(4.9) |
| **Ethnicity** |  |  |  |
| Not HISPANIC or LATINO |  |  | 917(83.6) |
| HISPANIC or LATINO |  |  | 171(15.6) |
| Others |  |  | 9(0.8) |
| **Estrogen receptor status** |  |  |  |
| Positive |  |  | 874(79.7) |
| Negative |  |  | 209(15.1) |
| Not evaluated |  |  | 14(1.3) |
| **Progesterone receptor status** |  |  |  |
| Positive |  |  | 751(68.5) |
| Negative |  |  | 329(30.0) |
| Not evaluated |  |  | 17(1.5) |
| **Lymph node stage** |  |  |  |
| Axillary lymph node dissection alone |  |  | 331(30.2) |
| Sentinel node biopsy alone |  |  | 260(23.7) |
| Sentinel lymph node biopsy plus axillary dissection |  |  | 259(23.6) |
| Others |  |  | 247(22.5) |
| **Distant metastasis present** |  |  |  |
| No |  |  | 393(35.8) |
| Yes |  |  | 20(1.8) |
| Others |  |  | 684(62.4) |
| **Hormone Therapy** |  |  |  |
| Yes |  |  | 370(33,7) |
| No |  |  | 374(34.1) |
| Others |  |  | 353(32.2) |
| **Chemotherapy** |  |  |  |
| Yes |  |  | 401(36.6) |
| No |  |  | 363(33.1) |
| Others |  |  | 333(30.4) |
| **Immunotherapy** |  |  |  |
| Yes |  |  | 34(3.1) |
| No |  |  | 830(75.7) |
| Others |  |  | 233(21.2) |
| **Targeted Molecular therapy** |  |  |  |
| Yes |  |  | 64(5.8) |
| No |  |  | 754(68.8) |
| Others |  |  | 279(25.4) |
