# Supplementary figures and images for: Identification of a novel lipid metabolism-related gene signature within the tumour immune microenvironment for breast cancer
Source: Lipids Health Dis. 2022 May 13;21:43. doi: 10.1186/s12944-022-01651-9 (PMC9103058; doi:10.1186/s12944-022-01651-9)

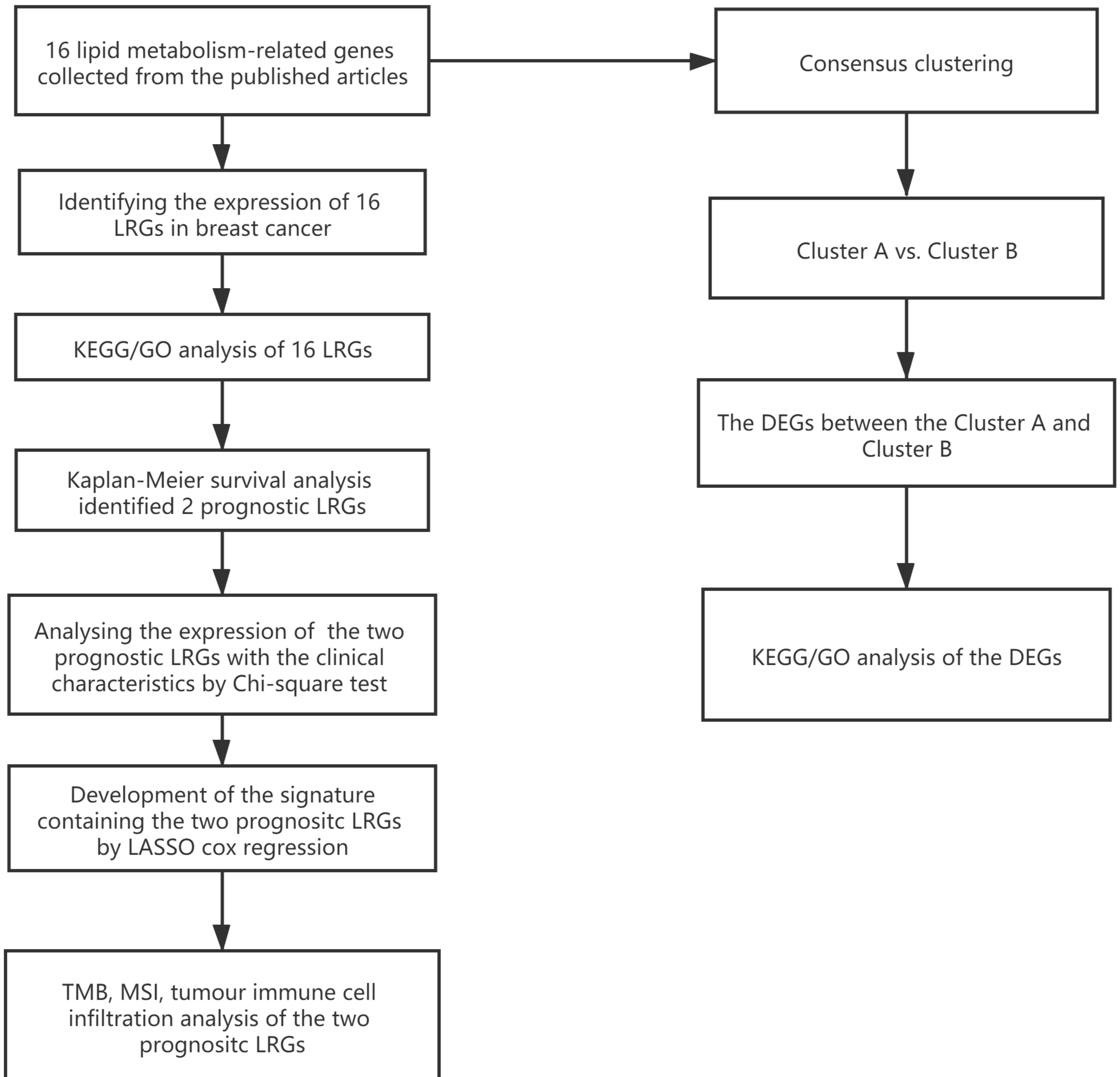

Supplement: Supplementary file 2 — Additional file 2. [file 12944_2022_1651_MOESM2_ESM.pdf]
